# Complete Genome Sequences of Three Outbreak-Associated *Legionella pneumophila* Isolates

**DOI:** 10.1128/genomeA.00696-16

**Published:** 2016-07-21

**Authors:** Shatavia S. Morrison, Heta P. Desai, Jeffrey W. Mercante, Pascal Lapierre, Brian H. Raphael, Kimberlee Musser, Jonas M. Winchell

**Affiliations:** a Division of Bacterial Diseases, National Center for Immunization and Respiratory Diseases, Centers for Disease Control and Prevention, Atlanta, Georgia, USA; b Wadsworth Center, New York State Department of Health, Albany, New York, USA

## Abstract

We report here the complete genome sequences of three *Legionella pneumophila* isolates that are associated with a Legionnaires’ disease outbreak in New York in 2012. Two clinical isolates (D7630 and D7632) and one environmental isolate (D7631) were recovered from this outbreak. A single isolate-specific virulence gene was found in D7632. These isolates were included in a large study evaluating the genomic resolution of various bioinformatics approaches for *L. pneumophila* serogroup 1 isolates.

## GENOME ANNOUNCEMENT

*Legionella pneumophila* is the primary etiologic agent of Legionnaires’ disease (LD) in the United States (1–3). *L. pneumophila* can be transmitted to a human host by inhalation of aerosolized water from a contaminated manmade water system. The majority of isolates in the CDC collection are serogroup 1 (sg 1), which are the primary cause of outbreaks (76.5% to 90%) (1, 3, 4). Here, we report the complete genome sequences of three *L. pneumophila* isolates, D7630, D7631, and D7632. These isolates are associated with an LD outbreak in the Bronx, NY, in 2012 (5). We designated isolates NY28, NY29, and NY30 as D7630, D7631, and D7632, respectively, all of which are sg 1 and sequence type 731 (ST731) (6, 7).

Each isolate was sequenced using the Illumina MiSeq (San Diego, CA) and Pacific Biosciences RSII (Menlo Park, CA) sequencing platforms. An average of 3,546,840 Illumina and 82,739 PacBio sequencing reads were used to construct completely closed genomes for each isolate. The *ab initio* gene finder algorithm Prokka (version 1.8), was used to predict 3,045, 3,038, and 3,036 coding sequences for D7630, D7631, and D7632, respectively, along with 43 predicted tRNAs for each isolate (8). Approximately 91.85% of the Illumina reads mapped to the *L. pneumophila* strain Philadelphia sg 1 reference (accession no. NC_002942) (9, 10). In a comparative analysis, 2,638 core genes were identified (11), representing approximately ~78.51% of the genome, which is similar to Gomez-Valero et al. (12). We identified 18, 5, and 1 isolate-specific gene for D7630, D7631, and D7632, respectively, the majority of which were classified as mobile elements or hypothetical proteins. All three isolates contained an *rtxA* gene that is 100% identical to that found in *L. pneumophila* strain Philadelphia. This gene is associated with *L. pneumophila* virulence, playing a key role in entry and replication within human macrophages (13–16). Interestingly, D7632 was found to contain an additional isolate-specific *rtxA* gene, which is highly similar to that from *L. pneumophila* Thunder Bay (91%). Further investigation is required to determine if this additional gene confers any growth or phenotypic advantages to this strain.

As whole-genome sequencing becomes a more readily available tool in public health laboratories for outbreak investigations, it is increasingly important to understand the genetic diversity of legionellae. Completely characterized sequences, such as those reported here, may help define relationships between outbreak- and nonoutbreak-related *L. pneumophila* isolates. An in-depth comparative genomics study will be performed in the future to define these relationships.

### Nucleotide sequence accession numbers.

This whole-genome shotgun project has been deposited at GenBank under accession numbers CP015344, CP015343, and CP015342 for D7630, D7631, and D7632, respectively.
